# Observational study of pimecrolimus 1% cream for prevention of transcutaneous sensitization in children with atopic dermatitis during their first year of life

**DOI:** 10.3389/fped.2023.1102354

**Published:** 2023-04-25

**Authors:** Nikolay N. Murashkin, Leyla S. Namazova-Baranova, Svetlana G. Makarova, Roman A. Ivanov, Stepan G. Grigorev, Dmitri V. Fedorov, Eduard T. Ambarchian, Roman V. Epishev, Alexander I. Materikin, Leonid A. Opryatin, Alena A. Savelova

**Affiliations:** ^1^ National Medical Research Center for Children’s Health, Moscow, Russia; ^2^ Sechenov First Moscow State Medical University (Sechenov University), Moscow, Russia; ^3^ Central State Medical Academy of the Presidential Administration of the Russian Federation, Moscow, Russia; ^4^ Research Institute for Pediatrics and Children’s Health Protection, Federal National Public Healthcare Institution “Central Clinical Hospital of the Russian Academy of Sciences”, Ministry of Science and Higher Education, Moscow, Russia; ^5^ Pirogov Russian National Research Medical University, Moscow, Russia; ^6^ Kirov Military Medical Academy, St Petersburg, Russia; ^7^ Pediatric Infectious Disease Clinical Research Center of the Federal Medical Biological Agency, St Petersburg, Russia

**Keywords:** atopic dermatitis, children, sensitization, food allergens, indoor allergens, pimecrolimus

## Abstract

**Introduction:**

Epidermal barrier dysfunction in children with atopic dermatitis can cause transcutaneous sensitization to allergens and allergic diseases. We evaluated the effectiveness of an early-intervention algorithm for atopic dermatitis treatment, utilizing pimecrolimus for long-term maintenance therapy, in reducing transcutaneous sensitization in infants.

**Method:**

This was a single-center cohort observational study that enrolled children aged 1-4 months with family history of allergic diseases, moderate-to-severe atopic dermatitis, and sensitization to ≥ 1 of the investigated allergens. Patients who sought medical attention at atopic dermatitis onset (within 10 days) were group 1 “baseline therapy with topical glucocorticoids with subsequent transition to pimecrolimus as maintenance therapy”; patients who sought medical attention later were group 2 “baseline and maintenance therapy with topical glucocorticoids, without subsequent use of pimecrolimus”. Sensitization class and level of allergen-specific immunoglobulin E were determined at baseline, and 6 and 12 months of age. Atopic dermatitis severity was evaluated using the Eczema Area and Severity Index score at baseline and 6, 9 and 12 months of age.

**Results:**

Fifty-six and 52 patients were enrolled in groups 1 and 2, respectively. Compared with group 2, group 1 demonstrated a lower level of sensitization to cow's milk protein, egg white and house dust mite allergen at 6 and 12 months of age, and a more pronounced decrease in atopic dermatitis severity at 6, 9 and 12 months of age. No adverse events occurred.

**Discussion:**

The pimecrolimus-containing algorithm was effective in treating atopic dermatitis and prophylaxis of early forms of allergic diseases in infants.

**Trial registration:**

https://clinicaltrials.gov/ NCT04900948, retrospectively registered, 25 May 2021.

## Introduction

Atopic dermatitis (AD) is a relapsing, chronic inflammatory skin disease characterized by pronounced itching (1). It affects both children and adults and has a deleterious impact on patients’ quality of life (1). In the Russian Federation, the prevalence of AD in different regions is between 6.2–15.5%, and two epidemiological analyses (one conducted between 2005 and 2010 and one conducted between 2010 and 2015) have demonstrated a 1.9-fold increase in AD prevalence during these time periods in the pediatric population of the Russian Federation (2).

AD commonly occurs in early childhood and can become a starting point of the atopic march, which is a typical sequence of development of allergic diseases, such as food allergy (FA), asthma and allergic rhinitis (3). One of the earliest stages in the development of allergic diseases and antigen sensitization is the emergence of a FA against the background of AD (4–6).

It is increasingly recognized that the development and uncontrolled course of AD in young children results in a higher risk of FA owing to epidermal barrier dysfunction and development of transepidermal sensitization, leading to a pathological immune response (7, 8). Normally, upon passage of food proteins through the gastrointestinal tract, antigen sensitization does not develop due to oral tolerance mediated by a tolerogenic population of gastrointestinal dendritic cells and production of regulatory Treg cells by the gastrointestinal tract's immune system (9). However, transcutaneous allergen passage in the case of epidermal barrier dysfunction may lead to sensitization and development of allergic reactions (10). The mechanism of transcutaneous sensitization with development of FA and immediate or delayed hypersensitivity reaction is complex. Its underlying pathogenesis involves penetration of an antigen through the epidermal barrier, leading to production of proinflammatory cytokines and chemokines, a Th2-type immune response and generation of allergen-specific immunoglobulin E (IgE) (11–14).

There is a need for both specific treatment and prophylactic measures to prevent transcutaneous sensitization and formation of FA/other allergic diseases accompanied by epidermal barrier dysfunction in children with AD. Studies suggest that emollients used straight after birth are an effective and safe method for AD prevention, which may reduce occurrence of allergic disease (6), although a recent meta-analysis suggests that this effect may delay rather than prevent AD (15). Furthermore, antihistamine therapy in certain at-risk groups may reduce the probability of asthma in children with AD in whom asthma has an allergic component (16, 17). However, the role of anti-inflammatory agents, including topical calcineurin inhibitors (TCI), in reducing risk of allergic diseases and preventing the atopic march due to transcutaneous sensitization in young children with AD remains unclear (16, 18).

As the pivotal factor in transcutaneous sensitization in AD is epidermal barrier dysfunction, treatment should aim to restore the skin barrier. Current guidelines for AD recommend topical glucocorticoids (tGC) as first-line therapy for reducing inflammation, with subsequent transition to maintenance therapy using TCI until complete resolution and to extend the remission period (19). Compared with tGC, the TCI pimecrolimus 1% cream (PIM) has a more specific anti-inflammatory and immunosuppressive mechanism of action (20, 21). In addition, PIM, unlike tGC, does not cause skin atrophy (22, 23), and is recommended for sensitive skin areas (19, 23). Evidence also indicates that PIM restores the epidermal barrier in patients with AD by altering expression of genes responsible for the normal structure and functioning of the skin barrier (24, 25). There is consensus among experts that off-label use of PIM is preferable in the treatment of children under 2 years of age because of its more favorable tolerability profile (23); indeed, a 5-year study showed that PIM was effective and had a favorable long-term safety profile when used in children with AD under 2 years of age (26). It should be noted that in the Russian Federation, PIM is approved for use from the age of 3 months (27).

The purpose of this cohort observational study was to evaluate an early-intervention algorithm for topical treatment of AD utilizing PIM as long-term maintenance therapy, aimed at prevention of transcutaneous sensitization and allergic diseases in children during their first year of life.

## Materials and methods

### Study design

This was a single-center cohort observational study (NCT04900948). The study was conducted at the Federal National State Institution “National Medical Research Center for Children's Health” of the Ministry of Healthcare of Russia from December 2017 to April 2020.

**Figure 1:**
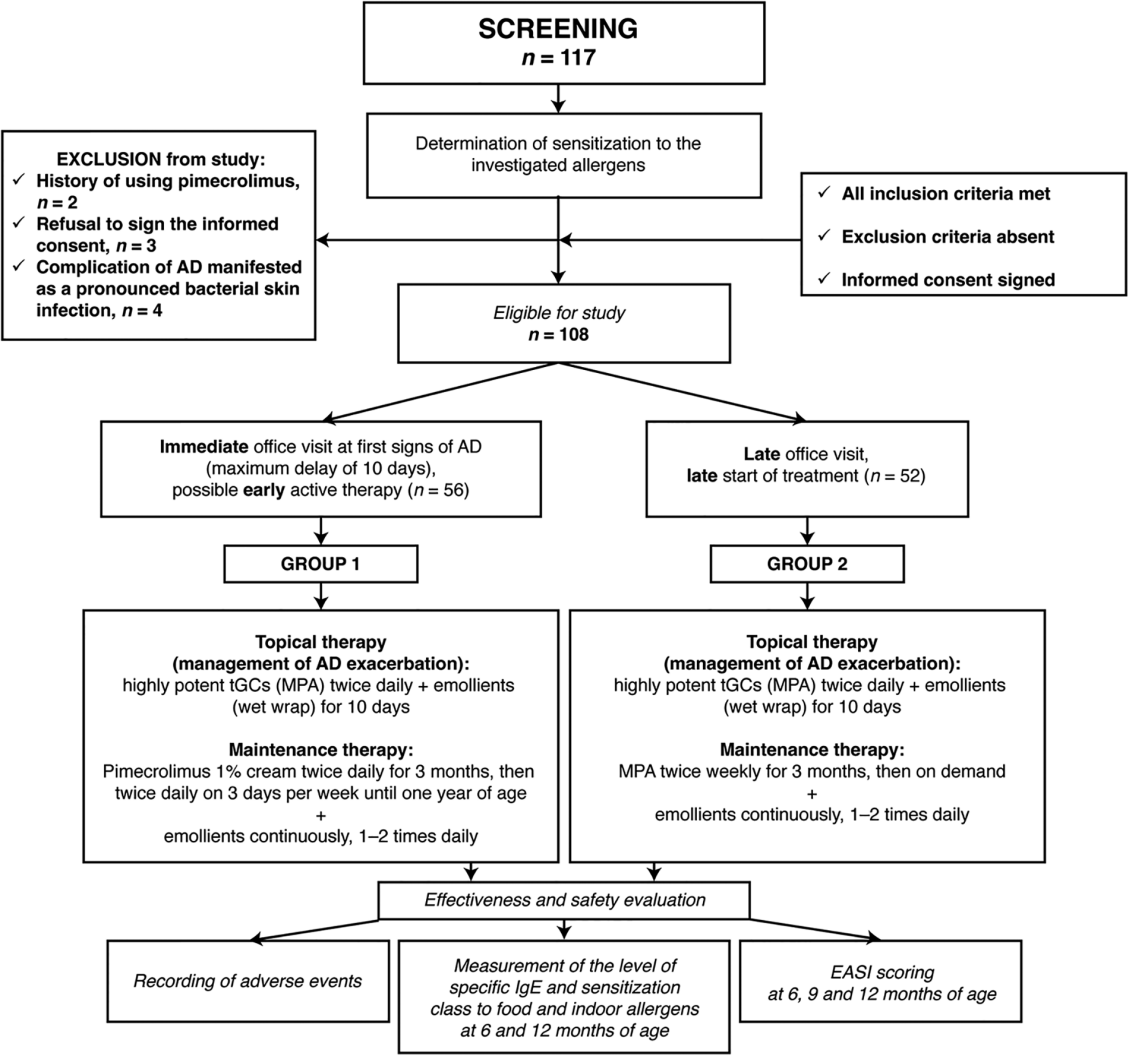
Study design flowchart. AD, atopic dermatitis; EASI, Eczema Area and Severity Index; tGC, topical glucocorticoids; MPA, methylprednisolone aceponate.

Patients were assigned to the groups according to the clinical situation and the time when they sought specialized medical attention: patients who sought medical attention immediately at the onset of AD (at the first signs of disease, with a maximum delay of 10 days) were included in group 1 “baseline therapy with tGC with subsequent transition to PIM as maintenance therapy according to the suggested regimen”; patients who sought medical attention later were included in group 2 “baseline therapy and maintenance therapy with tGC, without subsequent use of PIM” (Figure 1).

### Eligibility criteria

Patients were eligible for enrollment if they met the following inclusion criteria: aged 1–4 months; early onset of AD; Eczema Area and Severity Index (EASI) score ≥12; moderate or severe AD; a family history of allergies (presence of the following conditions in ≥1 parent: AD, FA, atopic asthma, allergic rhinitis); and sensitization to one or several of the investigated food and indoor allergens assessed by ImmunoCAP™ at the screening stage (cow's milk protein [CMP], egg white, house dust mite allergen [*Dermatophagoides pteronyssinus*], soy, wheat).

Patients were excluded if they had been treated with TCI (PIM) less than 30 days prior to enrollment; had a history of concomitant severe neurological, endocrine, cardiovascular, hepatic or renal diseases; or if acute bacterial or viral infections were present at enrollment.

### Diagnostic criteria

The diagnosis of AD was based on clinical findings and medical history, and presence of three main, and at least three additional, diagnostic criteria (28). The severity of AD was evaluated using the EASI score (29, 30). The presence of sensitization to food and indoor allergens in a child was determined using ImmunoCAP. A family history of allergies was confirmed if ≥1 parent had AD, FA, atopic asthma, or allergic rhinitis.

### Treatment

All enrolled children with confirmed sensitization to one or several of the investigated allergens were recommended to follow a hypoallergenic lifestyle and prescribed a hypoallergenic diet and/or an individual elimination diet in accordance with the current clinical guidelines for the management of children with this pathology (31, 32). For instance, for breastfed children with sensitization to CMP, a hypoallergenic dairy-free diet was prescribed to the breastfeeding mother. If the child received formula or mixed nutrition, an extensively hydrolyzed milk protein formula or an amino acid-based formula was prescribed to the child in accordance with existing algorithms (31, 32).

#### Baseline therapy

Children from both groups were prescribed baseline therapy, which consisted of short-term treatment with a very potent topical tGC (methylprednisolone aceponate) twice daily combined with emollients (with wet wrap) for 10 days to relieve acute symptoms. This baseline therapy was also used to manage exacerbations when necessary.

#### Maintenance therapy

After the baseline therapy, patients from group 1 were switched to a TCI: PIM was applied twice daily for 3 months, then twice daily on 3 days per week until one year of age, with concomitant continuous daily use of emollients (1–2 times daily).

Following the baseline therapy, children from group 2 were switched to methylprednisolone aceponate twice weekly for 3 months, then to an on-demand regimen (exacerbation of skin pathology). Patients from group 2 also used emollients continuously (1–2 times daily). PIM was not used by patients in group 2.

### Outcomes

#### Main outcomes

The main outcomes were the level of allergen-specific IgE (kUA/L) and the sensitization class (I–VI) to CMP, egg white, house dust mite allergen, soya, and wheat. These parameters were evaluated during examination of patients in order to determine the presence of sensitization to food and indoor allergens at the time of enrollment/screening, then at 6 and 12 months of age. The suggested therapeutic algorithm using PIM was deemed effective if the levels of allergen-specific IgE and sensitization class decreased during treatment and were lower in group 1 compared with group 2.

#### Additional outcomes

AD severity was assessed using the EASI score; the assessment took place at the time of enrollment/screening, then at 6, 9 and 12 months of age. The suggested therapeutic algorithm using PIM was deemed effective if it resulted in a more favorable and long-term stable course of AD with an EASI score ≤7 (mild AD) in group 1 compared with group 2.

#### Safety

The following adverse events were routinely monitored at 6, 9, and 12 months of age: local skin reactions (irritation, pruritus and skin redness, eruptions, exfoliation, dry skin, swelling); viral/bacterial infection; onset of allergic reactions (urticaria, angioedema); skin discoloration (hypopigmentation, hyperpigmentation). In an emergency, parents/legal guardians contacted investigators directly.

### Measurement of outcomes

The presence of sensitization to food and indoor allergens was determined using an indirect immunofluorescence technique with the aid of the automated immunoassay analyzer ImmunoCAP250 (UniCAP® System, Thermo Fisher Scientific, formerly Phadia АВ), with the analytical sensitivity of 0.01 kUA/L. Sensitization to allergens was defined as a specific IgE concentration of >0.35 kUA/L in blood serum (according to the manufacturer's instructions). The between-assay coefficient of variation for the ImmunoCAP test system was 3.9–6.6% (according to the manufacturer's instructions). The class of sensitization was determined by the concentration of IgE: class I, 0.35–0.7 kUA/L; class II, 0.71–3.5; class III, 3.51–17.5 kUA/L; class IV, 17.51–50 kUA/L; class V, 50.01–100 kUA/L; class VI, >100 kUA/L.

The severity of AD was evaluated using the EASI score (29, 30). The severity of AD is recommended to be assessed using the following ranges on the EASI scale: 0, no signs of disease; 0.1–1.0, almost clear skin; 1.1–7.0, mild; 7.1–21.0, moderate; 21.1–50.0, severe; 50.1–72.0, very severe (29).

### Statistical procedures

#### Sample size calculation

The required sample size was not calculated in advance.

#### Statistical methods

Statistical analysis of the dynamics of the investigated parameters and their comparison between study groups was performed by multi-factor analysis of variance (ANOVA) using the STATISTICA™ software package version 10.0 (StatSoft, USA). The results are provided as tables and figures using the arithmetic mean. Variation of parameters was evaluated using the median, lower quartile (25th percentile, Q_1_), upper quartile (75th percentile, Q_3_) and interquartile range (Q_1_–Q_3_).

Quantitative values were compared in independent samples using least significant difference (LSD) test (ANOVA), and qualitative values were analyzed using Pearson chi-square test.

## Results

A total of 117 children were examined and 108 were eligible for the study (Figure 1). Based on the clinical situation and the time when they sought specialized medical attention, 56 children were assigned to group 1 (office visit at first signs of AD with a maximum delay of 10 days; baseline therapy followed by PIM) and 52 children to group 2 (late office visit; baseline therapy followed by tGC). The children in group 1 had a mean (standard deviation) age of 76 (11) days, EASI score of 33.3 (5.1) and delay in seeking medical attention of 4.8 (2.3) days (Table 1). The children in group 2 were slightly older (93 [14] days), had more severe AD at baseline (EASI score of 34.6 [3.7]) and a greater delay in seeking medical care (33.9 [9.1] days) (Table 1). All patients completed the study.

**Table 1:** Baseline characteristics.

Parameter	Group 1 *n* = 56	Group 2 *n* = 52	*p* value
Mean age (SD), days	76 (11)	93 (14)	<0.001
Sex (male/female), *n*	31/25	28/24	0.872
Mean EASI score (SD)	33.3 (5.1)	34.6 (3.7)	0.133
Mean delay in seeking medical attention from the first clinical signs (SD), days	4.8 (2.3)	33.8 (9.1)	<0.001

EASI, Eczema Area and Severity Index; SD, standard deviation.

### Cow's milk protein

**Figure 2:**
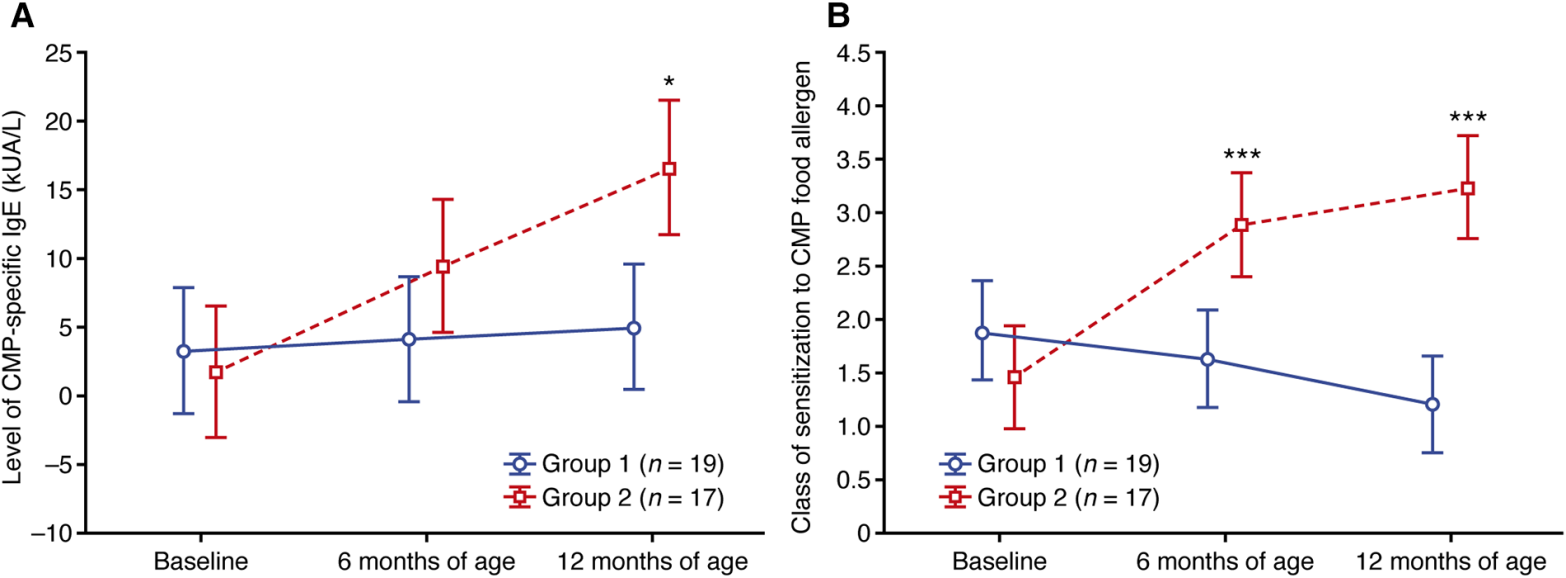
Change in (**A**) the level of CMP-specific IgE and (**B**) the class of sensitization to the CMP food allergen. Data shown are arithmetic mean ± 95% confidence interval. *p < 0.05; ***p < 0.001 vs. group 1. CMP, cow's milk protein.

Sensitization to CMP was found in 19 and 17 patients from groups 1 and 2, respectively (Supplementary Table S1). Although the levels of CMP-specific IgE were greater in group 1 compared with group 2 at baseline, the levels remained relatively stable (mean values; Figure 2A) or decreased (median values; Supplementary Table S1) at 6 and 12 months of age in group 1. In contrast, CMP-specific IgE levels increased from baseline to 6 and 12 months of age in group 2, with mean levels much greater in group 2 compared with group 1 at 12 months of age (16.5 vs. 5.0 kUA/L, respectively, *p *= 0.024; Figure 2A).

A similar pattern was observed for the class of CMP sensitization (both median and mean values), with decreases observed at 6 and 12 months of age relative to baseline in group 1 compared with increases at 6 and 12 months of age vs. baseline in group 2 (Supplementary Table S2; Figure 2B). By 12 months of age, the mean class of sensitization was 1.21 in group 1 and 3.24 in group 2 (*p *< 0.001; Supplementary Table S2; Figure 2B).

### Egg white

**Figure 3:**
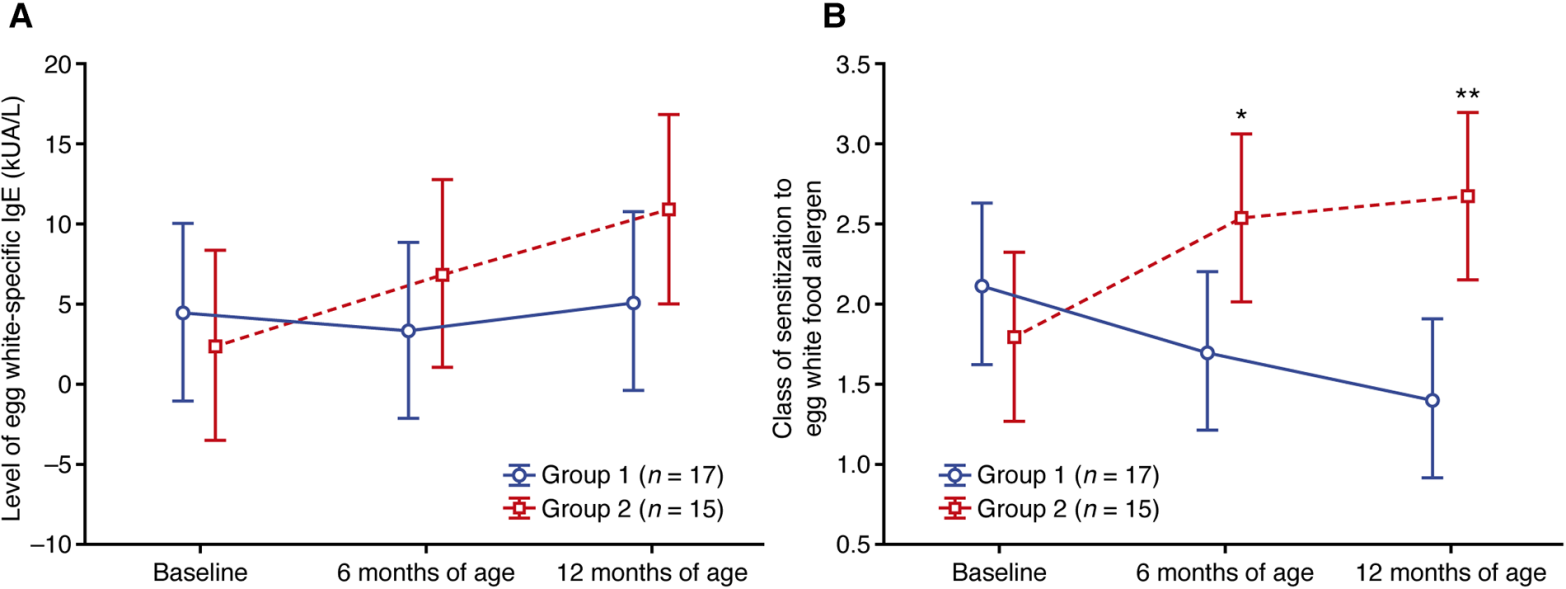
Change in (**A**) the level of egg white-specific IgE and (**B**) the class of sensitization to the egg white food allergen. Data shown are arithmetic mean ± 95% confidence interval. *p < 0.05; **p < 0.01 vs. group 1.

Sensitization to egg white was found in 17 and 15 patients from groups 1 and 2, respectively (Supplementary Table S3). Similar to CMP sensitization, higher mean and median levels of egg white-specific IgE were observed in group 1 at baseline compared with group 2 (Supplementary Table S3), and the levels in group 1 remained relatively stable (mean values; Figure 3A) or decreased (median values; Supplementary Table S3) at 6 and 12 months of age. In group 2, however, the mean and median levels of egg-white specific IgE increased at 6 and 12 months of age vs. baseline (mean values; Figure 3A).

Starting from 6 months of age, the class of egg white sensitization was lower in group 1 compared with group 2 (Figure 3B; Supplementary Table S4), with a mean class of 1.41 in group 1 and 2.67 in group 2 at 12 months (*p* = 0.007; Supplementary Table S4).

### House dust mite allergen

**Figure 4:**
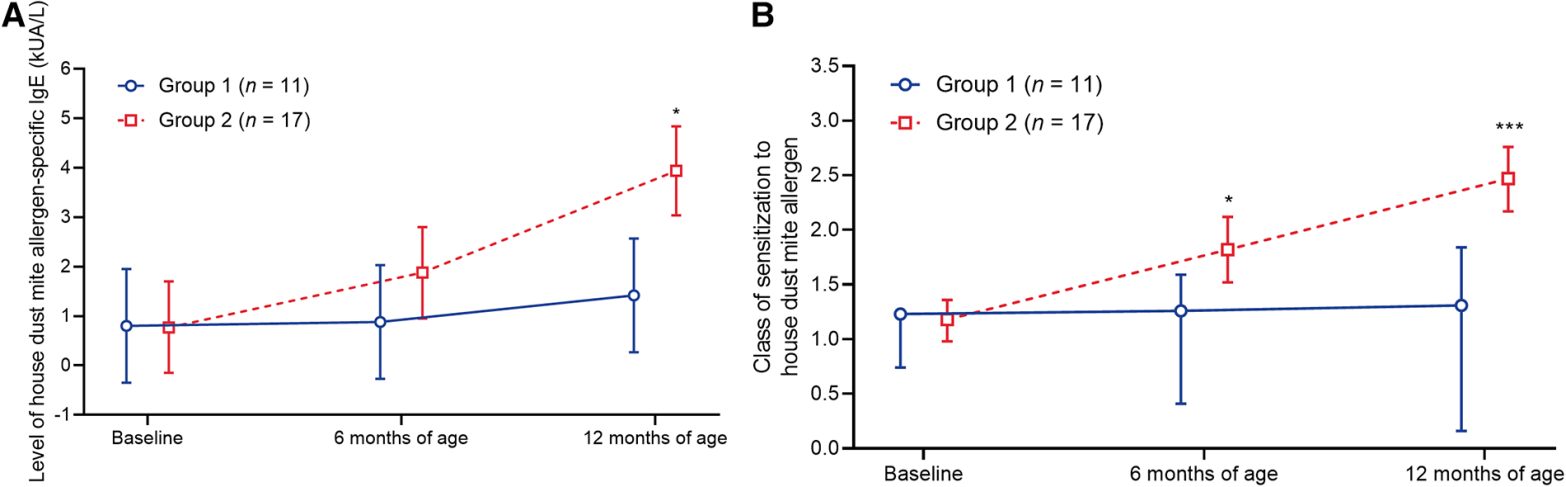
Change in (**A**) the level of house dust mite allergen-specific IgE and (**B**) the class of sensitization to the house dust mite allergen. Data shown are arithmetic mean ± 95% confidence interval. *p < 0.05; ***p < 0.001 vs. group 1.

Eleven patients from group 1 and 17 patients from group 2 were found to have sensitization to the indoor airborne allergen, house dust mite allergen (Supplementary Table S5). The levels of house dust mite allergen-specific IgE and class of house dust mite allergen sensitization remained relatively stable in group 1 throughout the study (Figure 4; Supplementary Tables S5, S6). In contrast, these parameters increased in group 2; both the level of house dust mite allergen-specific IgE and sensitization class were significantly greater in group 2 vs. group 1 at 12 months of age (Figure 4; Supplementary Tables S5, S6).

### Soya and wheat

**Figure 5:**
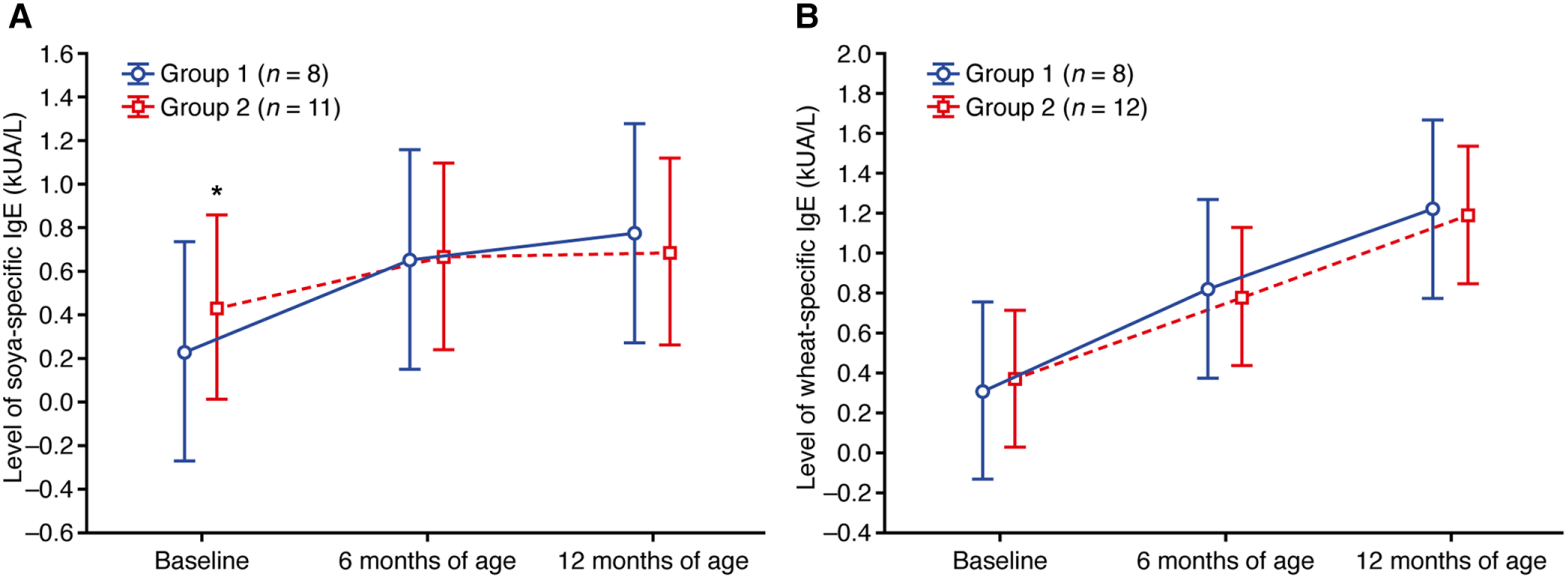
Change in the level of (**A**) soya-specific IgE and (**B**) wheat-specific IgE. Data shown are arithmetic mean ± 95% confidence interval. **p < 0.01 vs. group 1.

Within groups, baseline levels of soya- and wheat-specific IgE were similar (Figure 5; Supplementary Tables S7, S8). Mean levels of soya- and wheat-specific IgE stayed relatively stable in both groups up to 12 months of age (Figure 5; Supplementary Tables S7, S8).

### Severity of atopic dermatitis

**Figure 6:**
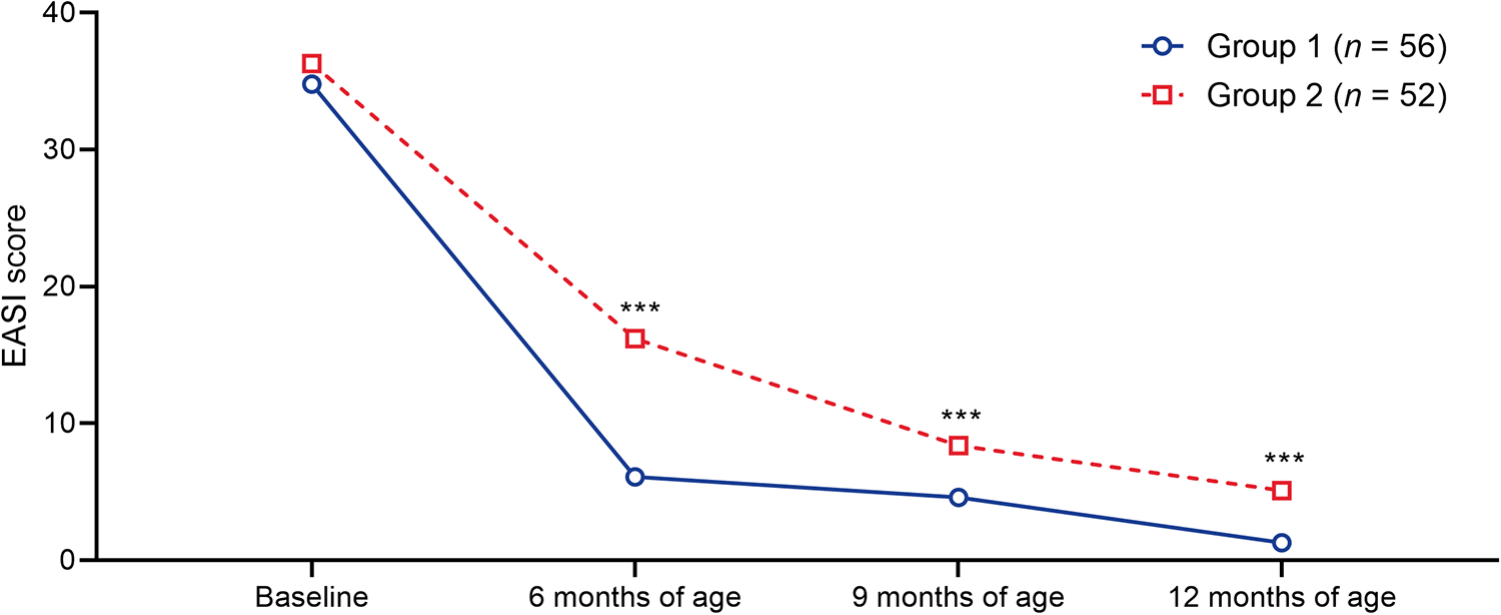
Timecourse of median EASI scores in the patient groups. ***p < 0.001 vs. group 1. EASI, Eczema Area and Severity Index.

There was a more rapid and pronounced improvement in the severity of AD in group 1 compared with group 2, represented by a decrease in EASI scores at 6, 9 and 12 months of age (Figure 6; Supplementary Table S9).

### Safety

No adverse events were reported.

## Discussion

The results of this study indicate that an algorithm using PIM for the treatment of AD, long-term maintenance therapy and prophylaxis of sensitization to food and indoor allergens was effective in preventing sensitization to CMP, egg white, and house dust mite allergen.

Group 1, which sought treatment early and received PIM as maintenance therapy, had lower values of the specific IgE level and sensitization class for CMP, egg white, and house dust mite allergen compared with group 2 (who received treatment later and did not receive PIM) at 6 and 12 months of age. Conversely, soya- and wheat-specific IgE levels were similar in group 1 and group 2 at baseline, 6 months, and 12 months of age, and IgE levels remained relatively stable throughout. This could be due to low baseline IgE levels and a low incidence of soya and wheat sensitization in the study participants. This is to be expected, considering the incidence of soya and wheat allergy in the general pediatric population is reported to be 0.5%, compared with 1.9% for milk (33).

Given that epidermal barrier dysfunction is a key factor in transcutaneous sensitization (11), the reduced levels of IgE and sensitization class for CMP, egg white, and house dust mite allergen in group 1 vs. group 2 could be due to effects of PIM on the skin barrier. Studies have shown that PIM restores the epidermal barrier in patients with AD by altering expression of genes responsible for synthesis of structural proteins that form part of a normal skin barrier (24, 25).

A previous study in children demonstrated that early treatment with PIM is associated with reduced incidence of major AD flares compared with vehicle (34). Similarly, our study suggests that early treatment with PIM is associated with a less severe course of AD. In addition, the effects of our PIM treatment algorithm on sensitization to food and indoor allergens indicate that the earlier treatment with PIM begins, the faster skin barrier function is restored and the less the risk of percutaneous allergen penetration.

There is convincing evidence for a favorable long-term safety profile for PIM when used by children under 2 years of age (23). For example, the 5-year long periodic use of PIM in the Petite study did not result in any safety signals in the form of an increased risk of infections, lymphomas or skin malignancies (26). Furthermore, when applied topically the systemic effect of PIM is minimal, likely due to the high molecular weight and lipophilicity of the compound (35, 36). The results of the current study support the safety profile for the long-term use of PIM, with no adverse events reported during treatment.

Although tGC are currently the first-line treatment of choice in inflammatory skin conditions (19), PIM has a more specific anti-inflammatory and immunosuppressive mechanism of action, exerting an effect on T lymphocytes and mast cells and thus minimizing skin atrophy (20, 21). As previously discussed, in contrast to corticosteroids, PIM has been shown to restore the epidermal barrier in patients with AD (24, 25). Together, these facts suggest that PIM may be considered for a more prominent role in the long-term management of such skin diseases (37).

### Limitations

A limitation of this study is that it was conducted at a single center in Russia with a relatively small sample size. However, we considered the patient population to be sufficient to draw general conclusions about the efficacy and safety of the treatment algorithms. Further study of PIM is warranted in larger patient populations to confirm our findings. As the patients in group 1 sought medical attention within 10 days of the onset of AD (mean delay 4.8 days) whereas patients in group 2 sought medical attention later (mean delay 33.8 days), the possibility that the effects of the PIM-containing treatment algorithm on sensitization to allergens and AD severity are primarily driven by early intervention rather than PIM cannot be excluded. Another limitation of the study includes the small number of food and indoor allergens investigated. While it was not within the scope of this study to investigate an exhaustive list of food and indoor allergens, future research should investigate use of pimecrolimus for the prevention of transcutaneous sensitization of other important allergens. Moreover, for clinical purposes, this study employed an open food challenge to confirm or rule out FAs, rather than an oral food challenge. Future studies are warranted investigating the effects of PIM using an oral food challenge, the gold standard for diagnosing FAs (38). Finally, calculation of the severity index on the EASI scale was carried out by several researchers, which may have led to distortions and differences in the results due to the subjectivity of the assessment.

## Conclusions

The results of this study indicate that an early-intervention algorithm that utilizes PIM for long-term maintenance therapy reduces the development of transcutaneous sensitization, thus preventing the formation and progression of allergic diseases in children with AD, including FA. Maintaining skin barrier function, and ensuring an immediate suppression of inflammation, appear to be key aspects of AD treatment and prophylaxis of clinical manifestations of allergic diseases. Additional studies including larger numbers of patients are needed to confirm the benefits of PIM in this population.

## Data Availability

The raw data supporting the conclusions of this article will be made available by the authors, without undue reservation.
