# Supplementary material for: Observational study of pimecrolimus 1% cream for prevention of transcutaneous sensitization in children with atopic dermatitis during their first year of life
Source: Front Pediatr. 2023 Apr 25;11:1102354. doi: 10.3389/fped.2023.1102354 (PMC10167287; doi:10.3389/fped.2023.1102354)
Supplement: Supplementary file 1 [file Datasheet1.docx]

**SUPPLEMENTAL MATERIAL**

**Supplemental Table 1** Levels of CMP-specific IgE in the study groups.

| Study period | CMP-specific IgE (kUA/L) | | | | *p* value |
| --- | --- | --- | --- | --- | --- |
|  | **Median and  quartiles [Q_1_–Q_3_]** | | **Arithmetic mean** | |  |
|  | **Group 1 (*n* = 19)** | **Group 2**  **(*n* = 17)** | **Group 1**  **(*n* = 19)** | **Group 2**  **(*n* = 17)** |  |
| Baseline | 1.7  [0.69–2.80] | 0.64  [0.45–0.90] | 3.2 | 1.8 | 0.339 |
| 6 months of age | 0.63  [0.42–3.70] | 7.50  [4.50–14.20] | 4.1 | 9.4 | 0.053 |
| 12 months of age | 0.40  [0.28–3.20] | 11.9  [7.70–17.80] | 5.0 | 16.5 | 0.024 |

CMP, cow’s milk protein.

**Supplemental Table 2** Class of sensitization (I–VI) to the CMP food allergen in the study groups.

| Study  period | Class of sensitization to CMP (I–VI) | | | | *p* value |
| --- | --- | --- | --- | --- | --- |
|  | **Median and  quartiles [Q_1_–Q_3_]** | | **Arithmetic mean** | |  |
|  | **Group 1  (*n* = 19)** | **Group 2  (*n* = 17)** | **Group 1 (*n* = 19)** | **Group 2  (*n* = 17)** |  |
| Baseline | 2  [1–2] | 1  [1–2] | 1.89 | 1.47 | 0.155 |
| 6 months of age | 1  [1–3] | 3  [3–3] | 1.63 | 2.88 | < 0.001 |
| 12 months of age | 1  [0–2] | 3  [3–4] | 1.21 | 3.24 | < 0.001 |

CMP, cow’s milk protein.

**Supplemental Table 3** Levels of egg white-specific IgE in the study groups.

| Study  period | Egg white-specific IgE (kUA/L) | | | | *p* value |
| --- | --- | --- | --- | --- | --- |
|  | **Median and  quartiles [Q_1_–Q_3_]** | | **Arithmetic mean** | |  |
|  | **Group 1  (*n* = 17)** | **Group 2  (*n* = 15)** | **Group 1  (*n* = 17)** | **Group 2  (*n* = 15)** |  |
| Baseline | 1.9   [0.96–3.10] | 0.90   [0.45–1.40] | 4.48 | 2.45 | 0.331 |
| 6 months of age | 0.82   [0.57–6.30] | 2.50   [1.60–5.60] | 3.35 | 6.90 | 0.328 |
| 12 months of age | 0.50   [0.29–2.80] | 2.60   [1.80–5.90] | 5.17 | 10.87 | 0.321 |

**Supplemental Table 4** Class of sensitization (I–VI) to the food allergen egg white in the study groups.

| Study  period | Class of sensitization to egg white (I–VI) | | | | *p* value |
| --- | --- | --- | --- | --- | --- |
|  | **Median and  quartiles [Q_1_–Q_3_]** | | **Arithmetic mean** | |  |
|  | **Group 1  (*n* = 17)** | **Group 2  (*n* = 15)** | **Group 1 (*n* = 17)** | **Group 2  (*n* = 15)** |  |
| Baseline | 2  [2–2] | 2  [1–2] | 2.12 | 1.80 | 0.282 |
| 6 months  of age | 2  [1–3] | 2  [2–3] | 1.71 | 2.53 | 0.024 |
| 12 months  of age | 1  [0–2] | 2  [2–3] | 1.41 | 2.67 | 0.007 |

**Supplemental Table 5** Levels of house dust-specific IgE in the study groups.

| Study  period | House dust-specific IgE (kUA/L) | | | | *p* value |
| --- | --- | --- | --- | --- | --- |
|  | **Median and  quartiles [Q_1_–Q_3_]** | | **Arithmetic mean** | |  |
|  | **Group 1  (*n* = 11)** | **Group 2  (*n* = 17)** | **Group 1 (*n* = 11)** | **Group 2  (*n* = 17)** |  |
| Baseline | 0.57  [0.42–0.67] | 0.40  [0.45–0.68] | 0.80 | 0.77 | 0.928 |
| 6 months  of age | 0.42  [0.32–1.40] | 1.20  [0.60–2.20] | 0.88 | 1.88 | 0.153 |
| 12 months  of age | 0.30  [0.13–3.60] | 3.30  [1.60–5.50] | 1.42 | 3.94 | 0.020 |

**Supplemental Table 6**  Class of sensitization (I–VI) to the indoor airborne allergen house dust in the study groups.

| Study  period | Class of sensitization to house dust (I–VI) | | | | *p* value |
| --- | --- | --- | --- | --- | --- |
|  | **Median and  quartiles [Q_1_–Q_3_]** | | **Arithmetic mean** | |  |
|  | **Group 1**  **(*n* = 11)** | **Group 2**  **(*n* = 17)** | **Group 1 (*n* = 11)** | **Group 2**  **(*n* = 17)** |  |
| Baseline | 1  [1–1] | 1  [1–1] | 1.23 | 1.18 | 0.281 |
| 6 months  of age | 1  [0–2] | 2  [1–2] | 1.26 | 1.82 | 0.012 |
| 12 months  of age | 0  [0–3] | 3  [2–3] | 1.31 | 2.47 | < 0.001 |

**Supplemental Table 7** Levels of soya-specific IgE in the study groups.

| Study  period | Soya-specific IgE (kUA/L) | | | | *p* value |
| --- | --- | --- | --- | --- | --- |
|  | **Median and  quartiles [Q_1_–Q_3_]** | | **Arithmetic mean** | |  |
|  | **Group 1  (*n* = 8)** | **Group 2  (*n* = 11)** | **Group 1 (*n* = 8)** | **Group 2  (*n* = 11)** |  |
| Baseline | 0.22  [0.17–0.27] | 0.40  [0.30–0.51] | 0.23 | 0.44 | 0.002 |
| 6 months  of age | 0.36  [0.31–0.42] | 2.40  [0.30–0.50] | 0.66 | 0.67 | 0.973 |
| 12 months  of age | 0.42  [0.37–0.46] | 2.50  [0.35–0.70] | 0.78 | 0.69 | 0.835 |

**Supplemental Table 8** Levels of wheat-specific IgE in the study groups.

| Study  period | Wheat-specific IgE (kUA/L) | | | | *p* value |
| --- | --- | --- | --- | --- | --- |
|  | **Median and  quartiles [Q_1_–Q_3_]** | | **Arithmetic mean** | |  |
|  | **Group 1  (*n* = 8)** | **Group 2  (*n* = 12)** | **Group 1 (*n* = 8)** | **Group 2  (*n* = 12)** |  |
| Baseline | 0.22  [0.17–0.27] | 0.38  [0.31–0.40] | 0.31 | 0.38 | 0.103 |
| 6 months  of age | 0.31  [0.57–0.42] | 0.68  [0.50–0.89] | 0.82 | 0.78 | 0.886 |
| 12 months  of age | 0.37  [0.36–0.46] | 0.90  [0.80–1.65] | 1.22 | 1.19 | 0.941 |

**Supplemental Table 9** EASI scores in the study groups.

| Study  period | EASI score  (median and quartiles [Q_1_–Q_3_]) | | *p* value |
| --- | --- | --- | --- |
|  | **Group 1  (*n* = 56)** | **Group 2  (*n* = 52)** |  |
| Baseline | 34.8  [28–38] | 36.3  [29–39] | 0.133 |
| 6 months of age | 6.1  [3–7] | 16.2  [13–22] | < 0.001 |
| 9 months of age | 4.6  [1–4] | 8.4  [5–11] | < 0.001 |
| 12 months of age | 1.3  [0–3] | 5.1  [2–6] | < 0.001 |

EASI, Eczema Area and Severity Index.
